# Learning Curve of the Application of Huang Three-Step Maneuver in a Laparoscopic Spleen-Preserving Splenic Hilar Lymphadenectomy for Advanced Gastric Cancer

**DOI:** 10.1097/MD.0000000000003252

**Published:** 2016-04-01

**Authors:** Ze-Ning Huang, Chang-Ming Huang, Chao-Hui Zheng, Ping Li, Jian-Wei Xie, Jia-Bin Wang, Jian-Xian Lin, Jun Lu, Qi-Yue Chen, Long-long Cao, Mi Lin, Ru-Hong Tu

**Affiliations:** From the Department of Gastric Surgery, Fujian Medical University Union Hospital, Fuzhou, Fujian Province, China.

## Abstract

To investigate the learning curve of the application of Huang 3-step maneuver, which was summarized and proposed by our center for the treatment of advanced upper gastric cancer.

From April 2012 to March 2013, 130 consecutive patients who underwent a laparoscopic spleen-preserving splenic hilar lymphadenectomy (LSPL) by a single surgeon who performed Huang 3-step maneuver were retrospectively analyzed. The learning curve was analyzed based on the moving average (MA) method and the cumulative sum method (CUSUM). Surgical outcomes, short-term outcomes, and follow-up results before and after learning curve were contrastively analyzed. A stepwise multivariate logistic regression was used for a multivariable analysis to determine the factors that affect the operative time using Huang 3-step maneuver.

Based on the CUSUM, the learning curve for Huang 3-step maneuver was divided into phase 1 (cases 1–40) and phase 2 (cases 41–130). The dissection time (DT) (*P *< 0.001), blood loss (BL) (*P *< 0.001), and number of vessels injured in phase 2 were significantly less than those in phase 1. There were no significant differences in the clinicopathological characteristics, short-term outcomes, or major postoperative complications between the learning curve phases. Univariate and multivariate analyses revealed that body mass index (BMI), short gastric vessels (SGVs), splenic hilar artery (SpA) type, and learning curve phase were significantly associated with DT. In the entire group, 124 patients were followed for a median time of 23.0 months (range, 3–30 months). There was no significant difference in the survival curve between phases.

AUGC patients with a BMI less than 25 kg/m^2^, a small number of SGVs, and a concentrated type of SpA are ideal candidates for surgeons who are in phase 1 of the learning curve.

## INTRODUCTION

According to the 14th Japanese gastric cancer treatment guidelines,^1^ for advanced upper gastric cancer (AUGC), the splenic hilar lymph node (SHLN) should be removed in a D2 LN dissection. Recent studies have found that the spleen participates in regulating the circulation of blood and the immune and endocrine systems, and the effect of a spleen-preserving splenic hilar lymphadenectomy has been gradually recognized.^2,3^ With the development of the laparoscopic technique, the safety and feasibility of a laparoscopic spleen-preserving splenic hilar lymphadenectomy (LSPL) has been gradually accepted by surgeons. Since Hyung et al^4^ first reported on LSPL in 2008, Japanese scholars and our center have reported on this procedure.^5,6^ However, it takes a certain number of cases to master the skill, meaning that surgeons new to the procedure experience a learning curve. Determining the learning curve is important for formulating a training plan and increasing confidence. The cumulative sum method (CUSUM) has been widely used to determine learning curves because of its ability to reflect the deviation of each data point from the mean value.^7–9^ However, the CUSUM has not yet been applied to the LSPL learning curve. Our center first performed LSPL in 2010.^10^ We had summarized an effective procedure for the performance of LSPL in clinical practice and divided the complex operation into 3 steps. Therefore, the operation was called Huang 3-step maneuver.^11,12^ The characteristics of Huang 3-step maneuver can help surgeons new to the procedure master the operation, but the learning curve is unclear. Therefore, in this study, we systematically evaluated the learning curve for applying Huang 3-step maneuver in LSPL for AUGC using the CUSUM.

## MATERIALS AND METHODS

### Study Population and Evaluation Parameters

**FIGURE 1:**
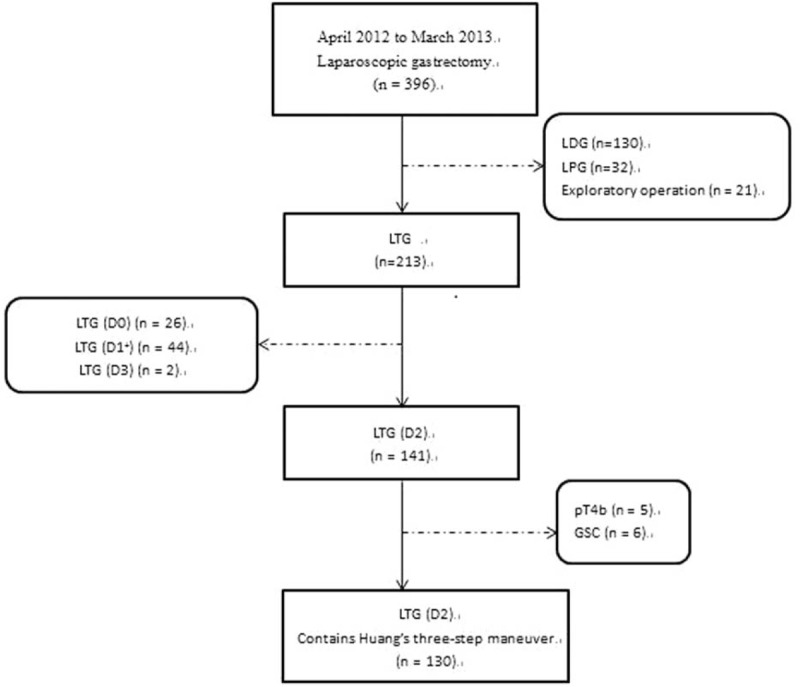
The flow chart of patient selection. GSC = gastric stump cancer, LDG = laparoscopic distal gastrectomy, LPG = laparoscopic partial gastrectomy, LTG = laparoscopic total gastrectomy.

A single surgeon (CMH) at Union Hospital, Fujian Medicine University performed a laparoscopic-assisted gastrectomy (LAG) on 396 consecutive patients between April 2012 and March 2013. Of this group, 213 patients underwent a laparoscopic total gastrectomy (LTG). For the patients who underwent an LTG (D0) (n = 26), LTG (D1+) (n = 43), or LTG (D3) (n = 2), the pathologic diagnosis was T4b (n = 5), and patients with remnant gastric cancer (GC) (n = 6) were excluded. Therefore, a total of 130 patients were included in this study (Figure 1). The surgeon performed his 1st LAG in May 2007 and his 1st LSPL in January 2010. After more than 100 cases of LSPL, he performed his 1st Huang 3-step maneuver in April 2012. The 1st assistant (CHZ) had assisted in more than 1000 LAGs. The advantages and disadvantages of the procedure were explained to the patients before surgery, and informed consent was provided both by the patients and their family members. The ethics committee of Fujian Union Hospital approved this retrospective study (Approval number: 20070428).

Dissection time (DT) was the time from dissecting the gastrosplenic ligament to dividing the last short gastric vessel (SGV). Blood loss (BL) was estimated by the volume of blood absorbed by the gauze and suctioned after subtracting the volume of fluids used for irrigation; the regional LNs dissected by Huang 3-step maneuver include LNs in the region of the splenic hilar (N0.10) and splenic hilar artery (SpA) trunk (N0.11d). When the SpA divided into its terminal branches <2 cm from the splenic hilum, it was considered to be concentrated. If the distance was ≥2 cm, it was considered to be distributed. The splenic lobar artery (SLA) refers to the terminal branch of the SpA at the splenic hilar and is divided into 4 types. If the SpA passes tortuously through the splenic hilar without dividing into terminal branches, it is the 1-branched type; if it branches off the superior and inferior SLAs, it is the 2-branched type; if it divides into the superior, middle, and inferior SLAs, it is the 3-branched type; and if it branches into 4 to 7 branches that enter the splenic hilar, it is the multiple-branched type.^13^ Postoperative major complications were considered severe morbidities if they occurred within 30 postoperative days and were higher than grade III (≥grade 3A) according to the Clavien–Dindo classification.^14^ Vascular injury was defined as intraoperative vascular injury bleeding due to the operation that required electric coagulation or titanium clip stanch.

Follow-up was performed by trained investigators through telephone calls, by recording the consultations of patients at the outpatient clinic, through mailings, or by visiting patients every 6 months. The follow-up period was through October 2014. The survival time was defined as the time from the surgical intervention to the last contact or date of death.

### Huang Three-Step Maneuver

The 1st step is the dissection of the LNs in the inferior pole region of the spleen. The 2nd step is the dissection of the LNs in the region of the SpA trunk. The 3rd step is the dissection of the LNs in the superior pole region of the spleen. Details of Huang 3-step maneuver were described in our previous study.^11,12^

### Statistical Analysis

All statistical analyses were performed using SPSS (Statistical Product and Service Solution 18 for Windows; SPSS Inc., Chicago, IL) except the moving average (MA) method and CUSUM plots, which were created in Excel 2010 (Microsoft, Redmond, Washington). The continuous data were reported as the mean ± SD and compared using a *t*-test. Qualitative data were compared using the Chi-square test or Fisher exact test.

To explore the factors affecting DT with Huang 3-step maneuver, the cases were divided into 3 groups according to DT quartiles. Group A included patients in the top 25% of DT (≤18 minutes), group B patients were between 25% and 75% of DT (18 < 29 minutes), and group C patients were in the bottom 25% of DT (≥29 minutes). We compared groups A and C with group B, and the variables with a *P* value <0.05 were selected for the multivariate stepwise logistic regression. The survival rate was calculated by the Kaplan–Meier method and compared using the log-rank test. *P* < 0.05 was considered statistically significant.

In this study, we analyzed DT and BL to evaluate the learning curve using the MA method and the CUSUM.

### MA Method

The MA method uses an average of subsets that are modified by adding new data and shifting all the datasets forward. The MA method can be used to confirm that a learning curve is associated with Huang 3-step maneuver. However, the MA typically lags, and when using the MA method to evaluate a learning curve, it has been suggested that the curve requires a longer period of time than what occurs in reality. In this study, the MA is defined as an average of DT or BL described below, where x_n_ is each DT or BL. In this study, an MA order of 20 was used. N indicates the case number. 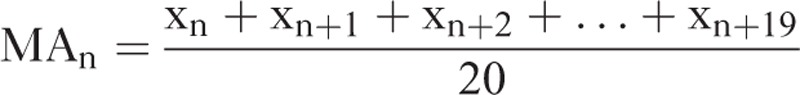


### CUSUM

The CUSUM is a statistical tool that can be used to evaluate small changes in specific cases and overall datasets. When a curve produced using the CUSUM has a downward slope, the mean of the data is decreasing. The division of the learning curve into 2 phases was based on results from the CUSUM. In this study, the CUSUM was applied using the following equation, where xi is an individual DT or BL, and μ is the mean of the overall DT or BL. N indicates the case number. 
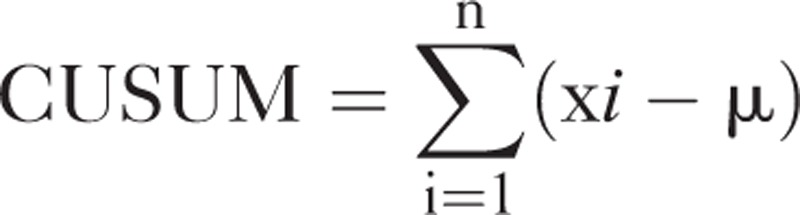


## RESULTS

### Overall Patient Characteristics

The 130 patients included 99 males (76.2%) and 31 females (23.8%). The mean DT was 23.5 ± 6.5 minutes, and the mean BL was 16.1 ± 6.6 mL. The average number of harvested SHLNs was 2.8 ± 2.4, and the average number of SHLN metastases was 0.18 ± 0.72. The postoperative pathological examination showed that the mean tumor size was 59.0 ± 30.5 mm (Table 1).

**TABLE 1:**
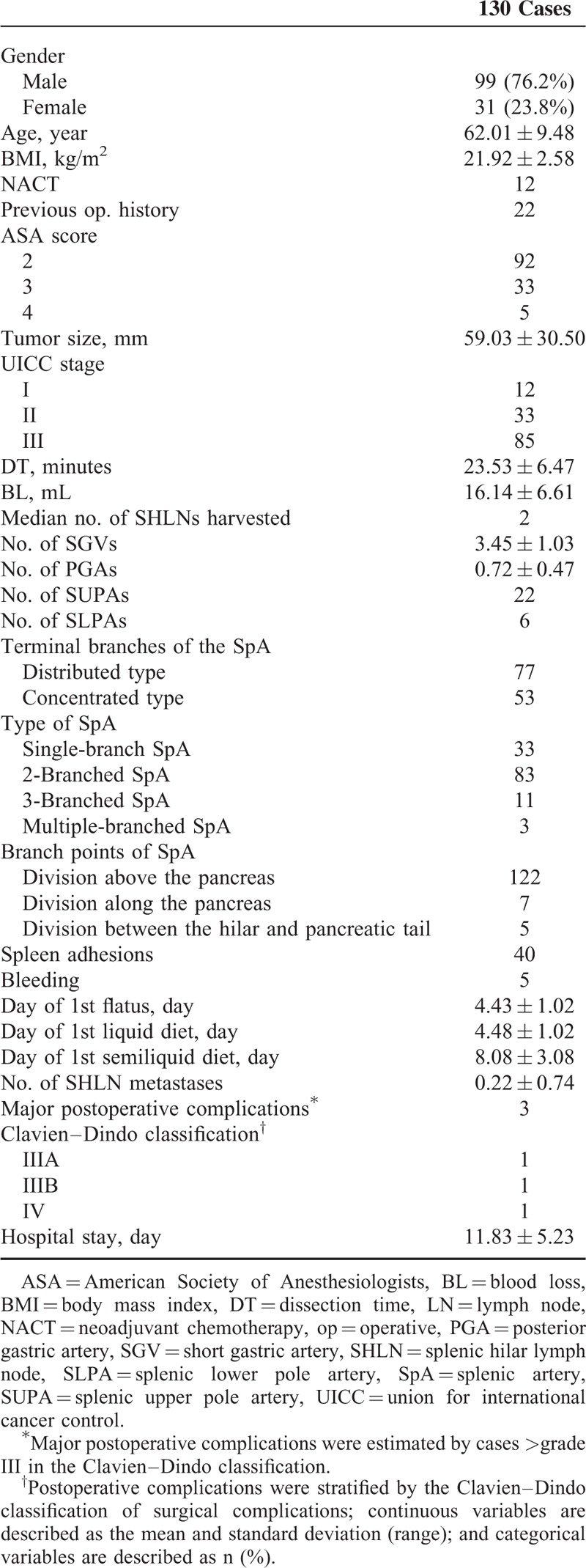
Patient Clinicopathological Characteristics

### Determining the Learning Curve

We researched the learning curve of Huang 3-step maneuver based on DT and BL.

**FIGURE 2:**
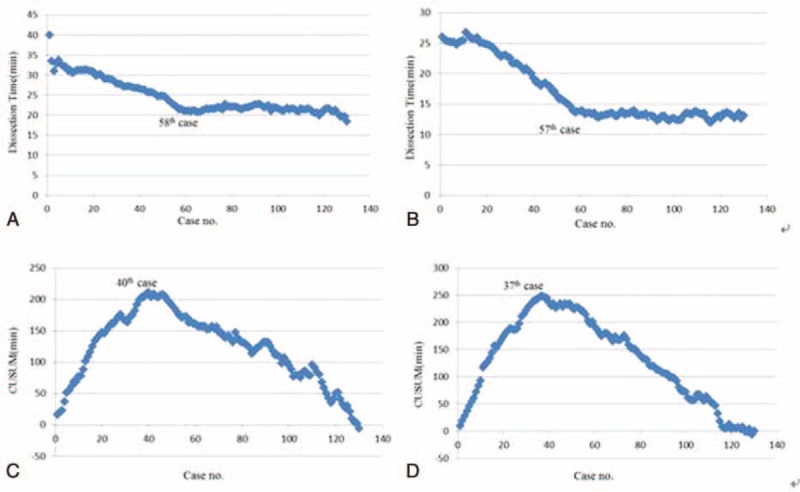
The MA method and CUSUM based on DT or BL. (A) The MA method based on DT; (B) the MA method based on BL; (C) the CUSUM based on DT; and (D) the CUSUM based on BL. BL = blood loss, CUSUM = cumulative sum method, DT = dissection time, MA = moving average.

The 1st step in recognizing the overall DT and BL trends was through the MA method. As shown in Figure 2A and B, with the accumulation of surgical cases, there was 1 low peak point in DT at the 58th case (21.55 minutes) and in BL at the 57th case (13.85 mL). Although the figure line has some small fluctuations after these points, it is relatively stable. In addition, considering the hysteresis effect of the MA method, it can forecast a learning curve before the 57th case.

The 2nd step in determining the learning curve period was through the CUSUM. According to the CUSUM, there were peak points in DT at the 40th case (211 minutes) (Figure 2C) and in BL at the 39th case (248.3 mL) (Figure 2D). Therefore, we concluded that the learning curve of Huang 3-step maneuver can be divided into 2 phases. There were inaccuracies when calculating BL,^15,16^ but DT was relatively accurate. Therefore, we selected DT to determine the learning curve in phase 1 (the initial learning period, cases 1–40) and phase 2 (the competent period, cases 41–130) in this study.

### Clinicopathologic Characteristics Compared Across Learning Phases

Gender, age, body mass index (BMI), and other factors showed no significant difference between the 2 learning phases (Table 2).

**TABLE 2:**
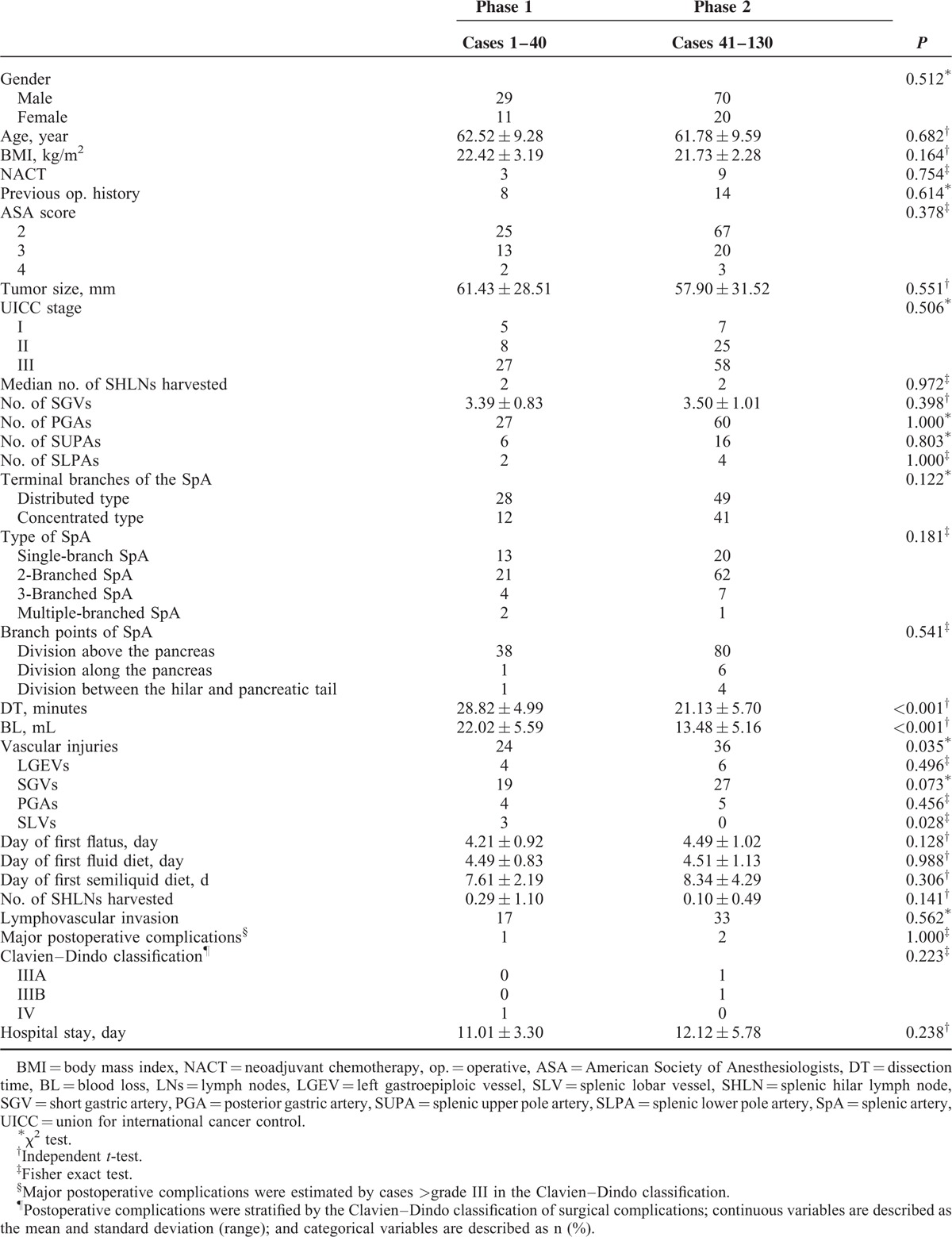
Clinicopathologic Characteristics and Perioperative and Pathologic Outcomes Stratified According to Learning Phase

### Perioperative Outcomes

DT and BL in phase 1 were significantly greater than those in phase 2 (*P* < 0.001). The number of intraoperative vascular injuries in phase 1 was also significantly greater than in phase 2. Among these injuries, the number of spleen lobe vessel (SLV) injuries in phase 1 was significantly higher than in phase 2 (*P* = 0.028). However, there were no differences in the number of injuries to the left gastroepiploic vessels (LGEVs), SGVs, or posterior gastric arteries between the 2 phases (*P* > 0.05). In addition, there were no significant differences among the day of 1st flatus, day of 1st liquid diet, day of 1st semiliquid diet, postoperative major complications, or length of hospital stay (Table 2).

### Pathologic Outcomes

There were no significant differences between the number of SHLNs harvested and the extent of histological and lymphovascular invasion (Table 2).

### Univariate and Multivariate Analyses of DT

The univariate analysis showed that a BMI ≥ 25 kg/m^2^, Union for International Cancer Control stage II, number of SGVs ≥3, distributed type SpA, and the phase 1 learning curve were closely related to DT. The multivariate analysis showed that BMI (*P* = 0.034), number of SGVs (*P* = 0.040), type of SpA (*P* = 0.020), and learning curve phase (*P* = 0.001) were independent, significant, and prognostic parameters of DT (Tables 3 and 4).

**TABLE 3:**
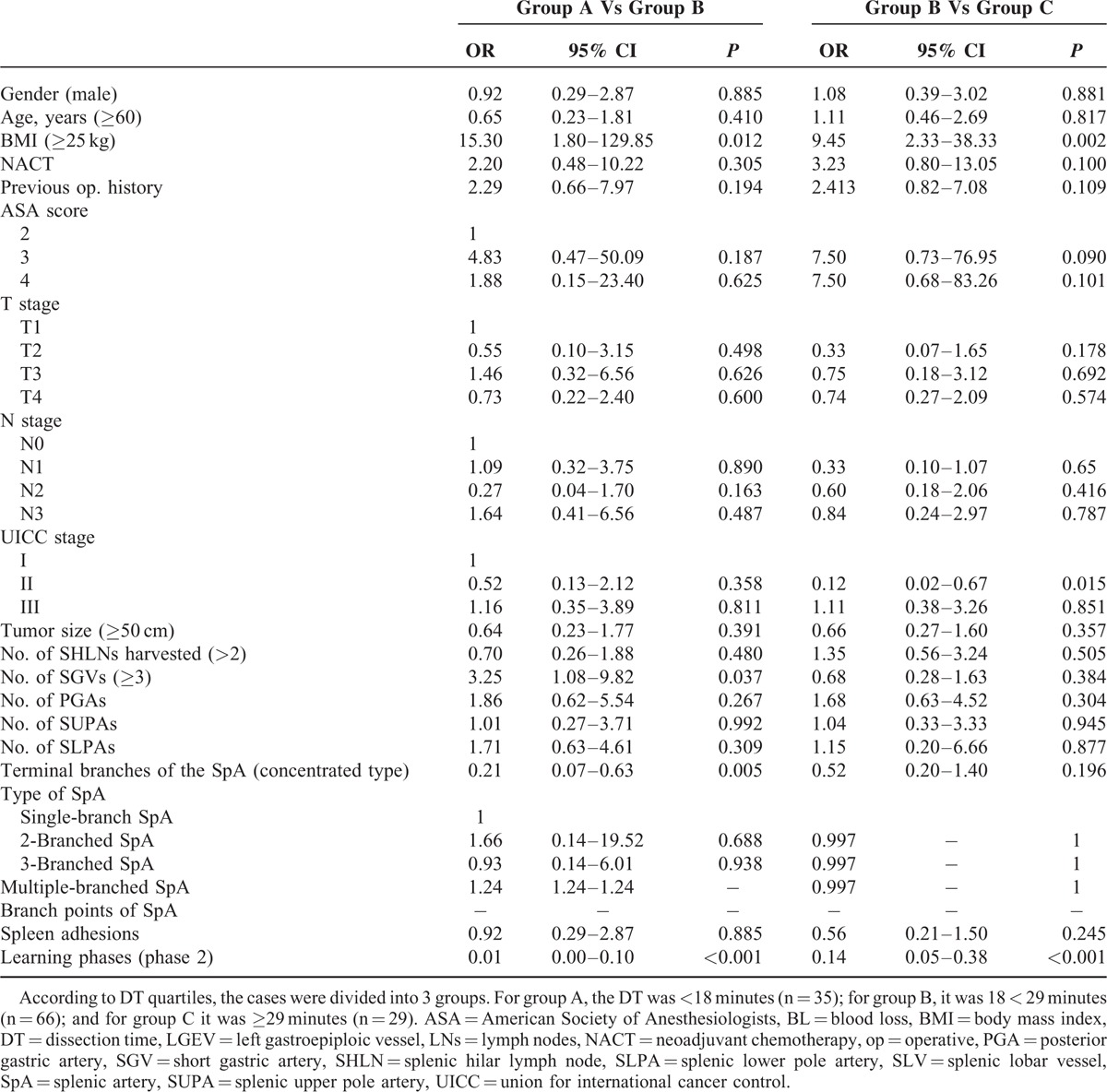
Univariate Analysis of the Factors That Impact Dissection Time

**TABLE 4:**
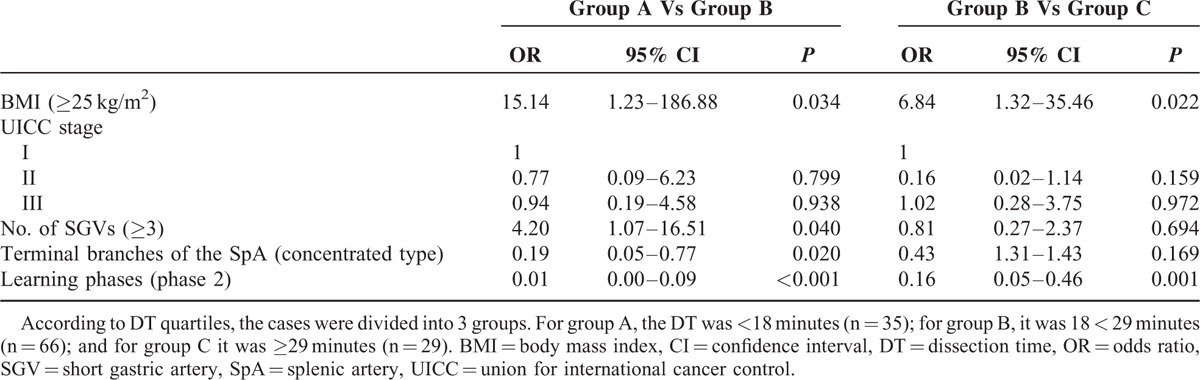
Multivariate Analyses of the Factors That Impact Dissection Time

### Survival Rates

**FIGURE 3:**
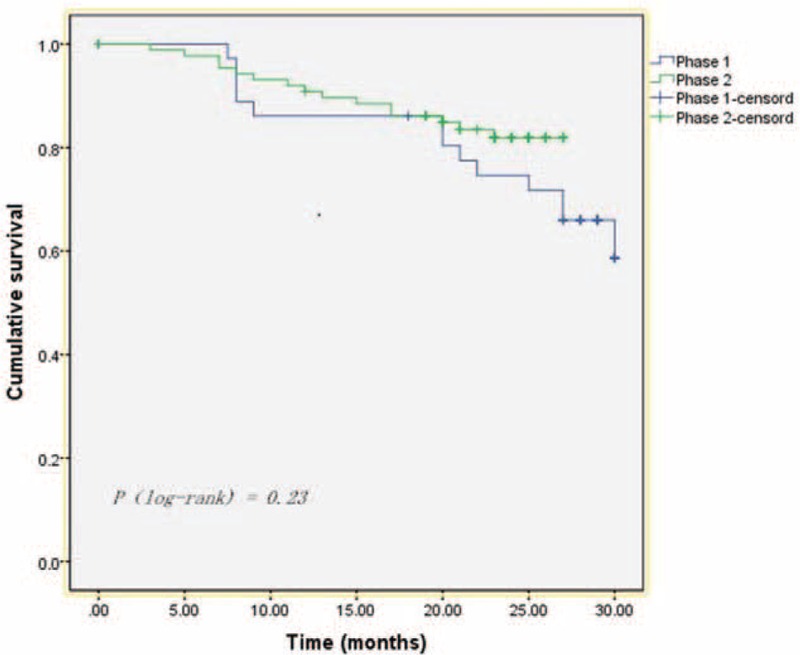
The cumulative survival curves of phase 1 and phase 2. Phase 1 = the initial learning period, cases 1 to 40; phase 2 = the competent period, cases 41 to 130.

The median follow-up for the entire cohort was 23.0 months (range, 3–30 months); in phases 1 and 2, the median follow-ups were 27 months (range, 8–30 months) and 22 months (range, 3–27 months), respectively. The follow-up rate was 95.4% and included 124 patients. There was no tumor recurrence or metastasis during the follow-up period, and there was no significant difference in the survival curve between the 2 phases (Figure 3).

## DISCUSSION

Previous studies showed a metastasis rate of SHLNs ranging from 9.8% to 34.4%.^17–20^ We previously evaluated 346 patients who underwent an LSPL for AUGC and found a metastasis rate of 10.1%.^21^ In addition, the 14th edition of the Guidelines for Gastric Cancer Treatment^1^ suggested that the SHLN should be removed via D2 lymph node dissection in cases of AUGC. However, because of the deep location of the splenic hilar, narrow operative space, fragile texture of the spleen, tortuosity of splenic vessels, and complicated branching of SLA, surgeons often need to free the spleen and pancreatic tail outside the abdominal cavity during open surgery. As the laparoscopic procedure has the advantages of long operative instruments, a magnified surgical view, and high visual resolution, the safety and effectiveness of LSPL has been established.^22^ However, a 2D view and the long operative instruments used in laparoscopic surgery require surgeons new to the procedure to become skilled through a step-by-step process, which entails a learning curve. Before the learning curve, surgeons need to practice and gain confidence so they can improve their reaction speed and reduce unnecessary procedures after the learning curve. Therefore, determining the learning curve is very important for developing a training plan, selecting appropriate cases, and establishing anticipated targets.

A previous study proved that the learning curves of various LAGs were different.^23–25^ Therefore, the learning curve of LSPL needs further research. Compared with our previous learning curve research, which used simple statistics,^26^ this study used the MA method and the CUSUM, which are more accurate statistical methods. The MA method calculates the mean value of a particular data point, which can eliminate the effects of cycle fluctuations and chance fluctuations in a single dataset. The advantage of using the MA method is its ability to observe long-term trends in data. The CUSUM monitors small changes in each data point to quickly analyze the continuous variation trend. Together, the 2 methods can accurately predict the development trend of an individual's learning curve and determine the point at which the skill has been mastered. We observed that the cutoff point of the learning curve of Huang 3-step maneuver was the 40th case, and there were significant difference in DT and BL before and after the cutoff point. Moreover, the number of vascular injuries before the cutoff point was significantly higher than after the cutoff point. This result proved that accumulated experience can improve the surgeon's skill through a deeper understanding of vascular anatomy and better collaboration with the team. Therefore, our result showing that a learning curve of 40 cases is accurate. In addition, the survival condition was similar between the phases, so surgeons with little or no experience with the procedure could perform it.

DT was the key factor in determining the learning curve in this study, and we studied the independent factors of DT to help beginners select appropriate cases and determine the fastest way through the learning curve. In a previous study, Hyung^27^ demonstrated that BMI is an independent factor that affects the operation time of a laparoscopic radical gastrectomy. He considered that a well-developed greater omentum and adipose tissue in the abdominal cavity of obese patients would affect the exposure of the surgical field and limit the operation space. In addition, because adipose tissue accumulates in the perivascular area and is very brittle, the area should be fully electrically coagulated to prevent bleeding, which increases the operation time. Based on this study, surgeons should separate and dissect more adipose tissue when dissecting the SHLNs of patients with a high BMI, which significantly increases DT. Variations in splenic hilar vessels also significantly affected DT. When dissecting SHLNs, surgeons often need to free SGVs to the root before they can divide them to avoid cutting the splenic lobar vessels (SLVs) and SGVs by mistake, which could lead to ischemia of the SLVs. With the distributed type of terminal branches of SpA, the SLVs are longer than in the concentrated type,^28^ and therefore, surgeons should denude longer vessels. In addition, the diameter of the SpA terminal branch in the concentrated type is much wider,^13^ which is advantageous for a lymphadenectomy because it can shorten DT. Therefore, it is beneficial for surgeons new to the procedure to select AUGC patients with a BMI less than 25, a small number of SGVs, and the concentrated type of SpA according to a radiological examination, such as 3D CT reconstruction and computed tomography. These surgeons will gain experience in successful cases, establish self-confidence, and shorten their learning curve. Although we utilized the CUSUM for data analysis, there are still several limitations to this study. First, we only evaluated the learning curve of a single surgeon; therefore, the universality of our results is not known. Second, the study used a retrospective, single-center design. A prospective, multiple-center study evaluating several surgeons would provide further insight into the learning curve associated with performing Huang 3-step maneuver.
